# Prospects of activity limitations among older adults in 23 low and middle income countries

**DOI:** 10.1038/s41598-020-67166-4

**Published:** 2020-06-26

**Authors:** Daniela Weber, Sergei Scherbov

**Affiliations:** 1 Wittgenstein Centre (IIASA, VID/ÖAW, WU), International Institute for Applied Systems Analysis (IIASA), Schlossplatz 1, 2361 Laxenburg, Austria; 2 Health Economics and Policy Division, Vienna University of Economics and Business, Welthandelsplatz 1, 1020 Vienna, Austria; 3 International Laboratory on Demography and Human Capital, Russian Presidential Academy of National Economy and Public Administration, Prospekt Vernadskogo, 84, 119571 Moscow, Russian Federation; 4 Vienna Institute of Demography, Austrian Academy of Science, Welthandelsplatz 2, 1020 Vienna, Austria

**Keywords:** Health care, Health policy

## Abstract

Increasing life expectancy and a growing share of older people around the world spotlight the issue of health during additional years of life. Research on trends of proportions of older people with activity limitations for low and middle income countries is sparse. We use data from the World Health Survey and the UN World Population Prospects to predict prevalence of activity limitations for 23 low and middle income countries for the upcoming 30 years. Our projections highlight huge variation in the proportion of older adults with limitations across investigated countries and this variation is not expected to diminish. However, these countries are facing considerable demographic changes and even though prevalence rates appear almost constant, absolute numbers are changing which require policy interventions. Furthermore, variations across countries reflect not only disparities in health conditions, but also differences in cultural peculiarities of reporting and historical perception of health.

## Introduction

For several decades upper-middle and high income countries are facing changes in their population compositions with shares of older adults increasing and shares of younger population declining. In many lower-middle and low income countries the proportion of young people remained high while life expectancy increased in particular within the last decade. Therefore, the proportion of unhealthy older adults may be expected to increase in those countries. However, additional life years are not necessarily spent with activity limitations but could also be spent in healthy conditions.

There are numerous studies on health trends in upper middle and high income countries. Studies on European countries and the United States showed an inconsistent pattern when it comes to health trends and disabilities. For instance, Jagger *et al*. identified only little changes in healthy life expectancy for Europeans^1^, while the trend in healthy life expectancy developed differently for the high and low educated population in the United States^2^. Lafortune and Balestat found similar results of an increasing, declining and even stable rates in disability among older adults from 12 OECD countries^3,4^. In the near future, the share of older adults with severe activity limitations in Europe is expected to remain constant, even though an increase in absolute numbers is expected^5,6^. These studies on upper middle and high income countries might guide some ideas about global future disability trends.

Research on health trends in low and middle income countries is sparse, although these countries are facing pronounced demographic changes in upcoming decades. A number of them, including China, the Russian Federation, and the Ukraine expect a considerable increase of the proportion of people at older ages^7–9^. Further, disability rates are higher in older age and moreover they are higher in low and middle income countries than in high income countries^10,11^. Studies show that the health status fell as the population grew older in low income countries in particular^12^ and that the total burden of disability increased by 52% between 1990 and 2017 due to aging population and noncommunicable diseases^13^. In Asia and the Pacific, fastest aging countries faced greatest increase in health dependencies^14^. Interestingly, in China disability prevalence is expected to decrease partly due to higher education and urbanization^15^. A study on Koreans aged at least 65 years also found a decline in disability rates during 1994 and 2004^16^. However, results depend on the metric of disability^17^ and thresholds (e.g. mild disabilities versus severe disabilities)^18^.

This paper looks at the health status based on activity limitations of older adults across the world and forecasts the share of older adults with severe activity limitations for the upcoming 30 years in 23 low and middle income countries. In particular, we focus on males and females above age 50 applying an innovative methodology of forecasting^5,19^.

## Results

### Disparities in activity limitations across countries

Data on self-reported activity limitations provide a very heterogenous picture across low and middle income countries. For instance, in China only 2.4% of the female population in the age group 60–69 years reported severe activity limitations, whereas about 69% of their counterparts in Morocco reported those limitations (Table 1). The variation in prevalence of conditions with activity limitations was slightly more narrow for males then for females for all age categories. Pronounced differences are also observed between sexes in some countries. In general, less men reported activity limitations than their female counterparts (Table 1). However, it is important to note, that pronounced differences in prevalence of severe activity limitations among selected countries might be to a large extent attributed to the different style of reporting subjective conditions of activity limitations.

**Table 1:** Observed prevalence rates for three selected 10-year age groups of women and men with activity limitations for 23 low and middle income countries in 2003 and the subsequent standard deviations (SD).

Country	50–59	60–69	70–79	50–59	60–69	70–79
	Women			Men		
Brazil	18.3	21.5	27.4	11.2	17.1	20.9
China	1.0	2.4	9.8	2.6	4.7	8.7
Croatia	8.8	19.3	35.8	14.1	11.0	33.5
Dominican Republic	8.9	8.6	37.9	10.9	8.1	18.7
Ecuador	11.7	14.3	20.9	4.8	7.1	11.6
Ethiopia	7.6	24.0	22.3	6.8	11.5	13.2
Georgia	20.6	33.3	40.1	15.5	19.3	35.5
Ghana	7.2	14.5	9.7	5.1	13.6	30.4
India	22.1	34.2	47.3	13.5	24.5	28.7
Kenya	9.7	12.2	31.2	7.0	15.7	17.5
Malaysia	2.4	4.5	9.0	1.7	5.1	8.0
Mauritius	20.7	17.4	32.4	10.4	13.3	19.3
Mexico	5.5	7.6	14.4	3.6	7.4	13.8
Morocco	42.2	69.0	57.8	31.1	56.5	52.6
Pakistan	5.6	19.5	29.8	6.6	8.0	25.5
Paraguay	6.3	7.2	13.2	1.7	3.8	14.9
Philippines	12.5	11.7	13.8	5.7	4.5	17.5
Russia	7.7	19.6	36.0	7.4	18.4	21.9
Sri Lanka	5.9	16.9	29.6	3.5	7.7	14.1
Tunisia	15.6	30.2	40.3	10.9	17.1	38.5
Turkey	24.7	34.3	40.0	9.0	17.1	19.7
Ukraine	11.6	42.1	34.0	4.6	15.5	33.0
Uruguay	4.6	4.6	2.7	2.6	2.5	2.8
SD	9.3	15.1	14.1	6.4	11.1	11.6

### Trends in ratio of activity limitations free life expactancy to life expectancy

Our random coefficient regression showed a decline in the ratio of activity limitations free life expectancy to life expectancy by age, as expected, and an overall smaller ratio for women than for men (Table 2). Moreover, the country estimates for men and women indicated a slightly bigger variation in *logit*(*r*) across countries for women with a variance of 0.53 and 0.49 for men (Table 2). More reproducible, we present estimates of the random country effects, which highlight the difference in country effects by gender with an SD of 0.73 for women and an SD of 0.71 for men (Table 3).

**Table 2:** Fixed effects estimates and country effect variances of the random coefficient model regressing the *logit*(*r*) considering random variation between men and women.

Fixed effects: estimates	Estimate	Std. Error	*Pr*(>*t*)
Intercept	2.59	(0.17)	***
age	−0.17	(0.36)	
*age*^*2*^	−2.51	(0.33)	***
sex (f)	−0.48	(0.07)	***
**Country effects: variances**			
Intercept	0.49		
Sex (f)	0.10		
Cov: country sex (f)	−0.03		
Residual variance	0.06		
AIC	177.79		
BIC	212.77		
Log Likelihood	−80.90		

***p < 0.0001, **p < 0.01, *p < 0.05

**Table 3:** Estimated random intercept (country effects) on the *logit*(*r*) for women and men.

Country	Women	Men	Country	Women	Men
Brazil	−0.34	−0.29	Mexico	0.54	0.48
China	0.95	0.89	Morocco	−1.56	−1.66
Croatia	−0.45	−0.41	Pakistan	−0.11	0.09
Dominican Republic	−0.03	−0.40	Paraguay	0.24	0.50
Ecuador	0.66	−0.05	Philippines	0.53	0.49
Ethiopia	0.27	0.27	Russia	−0.28	−0.26
Georgia	−0.79	−0.68	Sri Lanka	−0.12	0.28
Ghana	0.53	−0.06	Tunisia	−0.66	−0.86
India	−0.87	−0.50	Turkey	−0.74	−0.27
Kenya	−0.07	0.16	Ukraine	−0.28	−0.26
Malaysia	1.32	0.82	Uruguay	1.56	1.95
Mauritius	−0.28	−0.23			
			SD	0.73	0.71

### Forecasting severe activity limitations by 2047

Our projections show that the prevalence of 60+ year olds with severe activity limitations is expected to change very little in the next 30 years, among men showing a change of at most 2.1 percent points (Table 4) in particular. Thus, we can still expect about 50% of the male population above age 60 in Morocco to report severe activity limitations while we expect only about 2.6% of males in Uruguay to report similar conditions. The highest increase in the prevalence of activity limited adults can be expected for the population of females 60+ in Mauritius. It will increase from 27% in 2017 to 30% in 2047 (Table 4).

**Table 4:** Projected prevalence of at least 60 year old women and men with severe activity limitations in 2017, 2027, 2037, and 2047; in parentheses change since 2017.

Country	2017	2027	2037	2047	2017	2027	2037	2047
	Women				Men			
Brazil	27.53	27.56 (0)	28.35 (0.8)	29.04 (1.5)	18.64	18.7 (0.1)	19.11 (0.5)	19.35 (0.7)
China	9.71	9.59 (−0.1)	10.12 (0.4)	10.9 (1.2)	6.73	6.69 (0)	6.96 (0.2)	7.35 (0.6)
Croatia	33.57	33.5 (−0.1)	34.22 (0.6)	34.49 (0.9)	22.67	22.82 (0.1)	23.25 (0.6)	23.18 (0.5)
Dominican Republic	21.9	21.63 (−0.3)	22.14 (0.2)	22.74 (0.8)	20.80	20.54 (−0.3)	20.97 (0.2)	21.3 (0.5)
Ecuador	11.94	11.83 (−0.1)	12.13 (0.2)	12.5 (0.6)	14.94	15 (0.1)	15.31 (0.4)	15.5 (0.6)
Ethiopia	18.32	18.28 (0)	17.92 (−0.4)	17.34 (−1)	12.41	12.36 (−0.1)	11.99 (−0.4)	11.7 (−0.7)
Georgia	41.76	40.99 (−0.8)	41.94 (0.2)	42.26 (0.5)	28.69	27.94 (−0.8)	28.46 (−0.2)	28.44 (−0.2)
Ghana	15.49	14.88 (−0.6)	14.75 (−0.7)	14.8 (−0.7)	16.93	16.51 (−0.4)	16.43 (−0.5)	16.46 (−0.5)
India	41.31	41.42 (0.1)	41.71 (0.4)	41.66 (0.3)	23.10	23.34 (0.2)	23.6 (0.5)	23.6 (0.5)
Kenya	23.44	23.13 (−0.3)	22.69 (−0.8)	22.42 (−1)	13.28	12.98 (−0.3)	12.62 (−0.7)	12.59 (−0.7)
Malaysia	6.66	6.7 (0)	6.93 (0.3)	6.85 (0.2)	7.02	7.05 (0)	7.23 (0.2)	7.01 (0)
Mauritius	26.71	27.42 (0.7)	29.05 (2.3)	30 (3.3)	17.99	18.52 (0.5)	19.61 (1.6)	20.11 (2.1)
Mexico	13.5	13.03 (−0.5)	12.98 (−0.5)	13.74 (0.2)	9.26	9.21 (0)	9.09 (−0.2)	9.4 (0.1)
Morocco	58.47	57.89 (−0.6)	58.65 (0.2)	58.95 (0.5)	48.72	48.87 (0.1)	49.95 (1.2)	49.5 (0.8)
Pakistan	25.22	24.79 (−0.4)	24.88 (−0.3)	24.76 (−0.5)	14.69	14.36 (−0.3)	14.36 (−0.3)	14.35 (−0.3)
Paraguay	18.02	18.31 (0.3)	18.8 (0.8)	18.27 (0.2)	9.46	9.81 (0.3)	10.07 (0.6)	9.78 (0.3)
Philippines	14.55	14.44 (−0.1)	14.63 (0.1)	14.83 (0.3)	10.02	10.09 (0.1)	10.29 (0.3)	10.41 (0.4)
Russia	29.33	29.3 (0)	30.57 (1.2)	30.08 (0.8)	19.95	20.23 (0.3)	20.9 (0.9)	20.13 (0.2)
Sri Lanka	23.69	24.36 (0.7)	25.27 (1.6)	25.81 (2.1)	11.34	11.72 (0.4)	12.27 (0.9)	12.76 (1.4)
Tunisia	36.15	35.42 (−0.7)	36.31 (0.2)	36.66 (0.5)	30.35	29.72 (−0.6)	30.54 (0.2)	30.45 (0.1)
Turkey	37.46	37.27 (−0.2)	37.66 (0.2)	38.4 (0.9)	19.43	18.86 (−0.6)	18.81 (−0.6)	19.1 (−0.3)
Ukraine	30.03	29.88 (−0.2)	30.47 (0.4)	30.13 (0.1)	20.59	20.57 (0)	20.62 (0)	20.05 (−0.5)
Uruguay	6.18	6.05 (−0.1)	5.97 (−0.2)	6.14 (0)	2.69	2.6 (−0.1)	2.57 (−0.1)	2.61 (−0.1)

### Ages with similar prevalence

**Figure 1:**
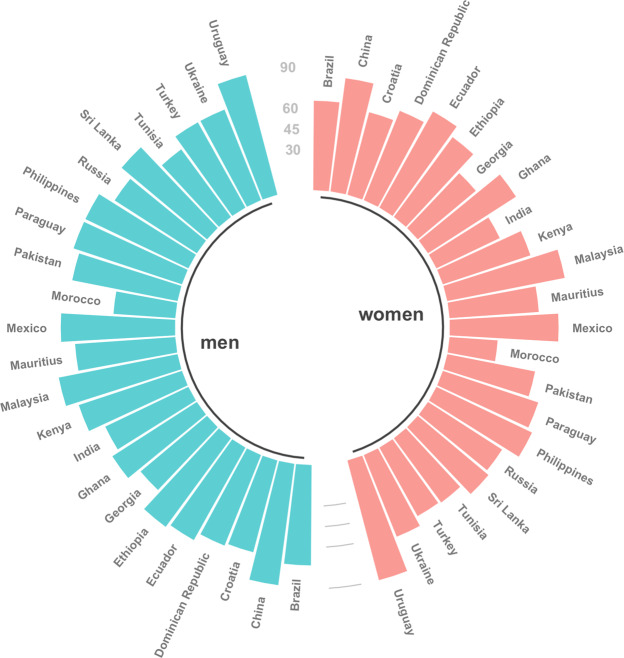
Men’s and women’s age at which 25% prevalence rate of living with severe activity limitations is attained in 2017 by country.

**Figure 2:**
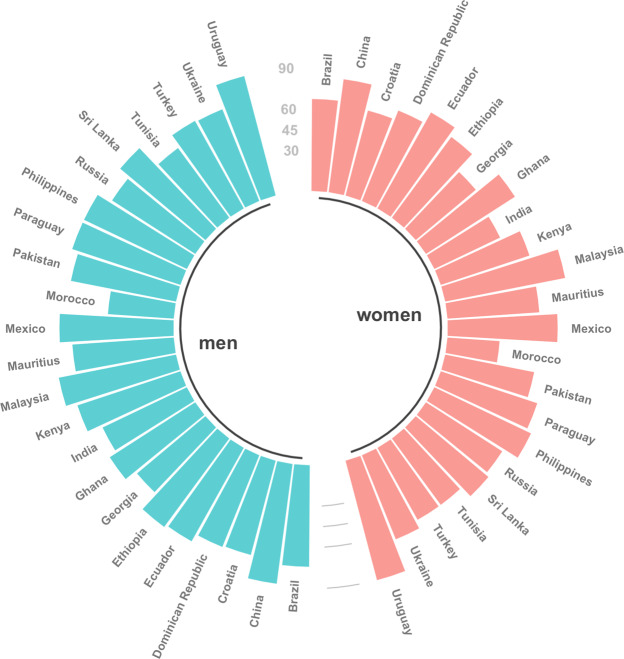
Men’s and women’s age at which 25% prevalence rate of living with severe activity limitations is attained in 2047 by country.

Our results also demonstrate a large variation across countries in comparing ages when a fixed level of prevalence is reached. We estimated the ages when 25% prevalence of activity limitations was attained. On average, every fourth woman was expected to report severe activity limitations at the age of only 51 years in India, 55 years in Georgia, and 57 years in Turkey in 2017. In contrast, every fourth woman in China, Ecuador, and Malaysia at the age of 80 and above reported activity limitations. Hence, they considered themselves having the same level of activity limitations at higher ages than their counterparts in India and Georgia at younger ages (Table 5, Fig. 1). This pattern won’t change much within the upcoming 30 years (Table 5, Fig. 2). Since at each age in most cases men report less activity limitations than women, it is not a surprise that 25% prevalence rate was reached by men at higher ages then by women. Nevertheless, we still find quite some differences across countries (Table 5, Fig. 1) with every fourth man in Tunisia and Georgia aged about 60 reporting activity limitations while the same rate was reached by men in Malaysia and China at about 90 years in 2017.

**Table 5:** The age at which 25% prevalence rate of living with severe activity limitations is attained in years 2017 and 2047 separately for women and men.

Country	2017	2047	2017	2047
	Women		Men	
Brazil	65	67	73	74
China	83	83	89	88
Croatia	62	64	69	71
Dominican Republic	70	71	71	72
Ecuador	80	80	77	78
Ethiopia	72	73	80	80
Georgia	55	57	63	65
Ghana	76	77	73	74
India	51	53	67	68
Kenya	67	68	79	79
Malaysia	87	89	88	87
Mauritius	65	67	73	74
Mexico	79	80	83	83
Morocco	<40	<40	45	48
Pakistan	65	66	78	78
Paraguay	73	74	83	83
Philippines	77	78	83	83
Russia	64	66	71	72
Sri Lanka	67	69	81	81
Tunisia	58	60	61	63
Turkey	57	60	71	73
Ukraine	64	66	71	72
Uruguay	>90	>90	>90	>90

## Discussion

In this analysis of WHO and United Nations data, we studied self-reports in activity limitations of older adults in 23 low and middle income countries. Our results showed that prevalence rates of older adults with severe activity limitations vary considerably across countries, as well as within countries between sexes.

Of course to a large extent this difference in reporting activity limitations reflects s not only health conditions in the countries, but also a different style of reporting which might be due to the cultural and historical perception of health^20–23^. However, different health measures show a varying magnitude in reporting bias^21^. Previous research on reporting mobility difficulties support our results showing significant cultural differences in self-reports of older adults from low- and middle income countries^20^. Further, differences across European countries in self-reported health measures suggested cultural differences in reporting styles in addition to differences in other national factors^5,19,24^. The reasons for cultural differences in reporting might be manifold. For instance, the threshold for reporting difficulties in activities might vary across cultures as it was shown for reporting difficulties in walking^25^. Moreover, culture influences the value of health, which corresponds with health promoting life styles shown for Taiwanese and American adolescents^26^. We further speculate that cultures with a higher value of health might also correspond to a lower threshold for reporting health limitations, thus this might lead to more reports than in other countries with a lower value of health and higher thresholds. Nevertheless, research on determinants of cultural differences in self-reports is sparse, for low and middle income countries in particular. Thus, we should not pay too much attention to the level of prevalence. Instead we should compare the dynamics of prevalence in each particular country.

Our major important finding shows for the first time that despite expected increases in life expectancy in all selected countries, the prevalence rate of people living with severe activity limitations is expected to change very little over time. However, the absolute number of people with activity limitations will still increase with the increase in older adults.

Previous studies have investigated trends in disabilities and activity limitations of older adults for high income countries such as Singapore, the United States and European countries^5,27–29^. Their findings were similar to our results on low and middle income countries expecting a constant prevalence rate, but an increase in absolute numbers.

In addition, we also highlighted the ages with the same prevalence rates as age might be a more common metric. When comparing ages with the same prevalence of severe activity limitations we observe considerable variations. For instance in 2017 79-year old women in Mexico demonstrate the same prevalence rate in activity limitations as their 51 year old counterparts in India. However, one should also be cautious in comparing different countries since the same argument that is mentioned above about different culture in reporting can be applied here. Further research on objective measures need to be conducted to exclude a potential cultural reporting bias.

## Methods

### Data source

In this study we used two different data sources: the World Health Organization’s World Health Survey and the United Nations World Population Prospects 2017. The World Health Survey (WHS)^30^ was conducted once in 2002–2004 to monitor the health outcomes of the population aged 18 years and above in more than 70 countries. We use the national representative survey data for low and middle income countries (World Bank classification 2003)^31^, which provide information on self-perceived activity limitations within the last month prior to their interview. The WHS includes the most recent available data enabling comparable international investigations on activity limitations, health and aging^32,33^. We excluded some countries due to sample size issues and reliability and ended up with a sample of 23 countries.

In addition to the WHS survey data source, we used sex specific abridged life tables for the period 2000–2050 from the United Nations^9^. Country specific population estimates and projected population trends were also retrieved from the United Nations World Population Prospects 2017. We used the population projections for 23 selected countries from the medium scenario by sex and 5-year age groups for the period 2015 to 2050.

### Health status

Our analysis is based on the responses to the WHS question related to self-perceived activity limitations. In the survey the question was asked: “Overall in the last 30 days, how much difficulty did you have with work or household activities?”. Participants could rate their response ranging from none, mild, moderate to severe, and extreme/ cannot do. We dichotomized the responses into *no severe limitations* (combining the response categories none, mild and moderate), and *severe limitations* (merging severe and extreme limitations). This health question is very similar to the well acknowledged Global Activity Limitation Indicator (GALI) except of the investigation period of one month instead of six months^34,35^. The validity and reliability of GALI is verified by several studies not only on an European population but also on an Asian population^36,37^.

### Statistical analysis

For each country we calculated the prevalence of people without severe activity limitations by 5-year age groups and for a first descriptive overview also by three selected 10-year age groups. Time of the survey was 2003. Sample weights were applied to account for non-response and to allow nationally representativeness for non-institutionalized population. We applied Sullivan’s method^38^ to compute activity limitations free life expectancy (HLE) for each country by sex and 5-year age groups and computed *r*, the ratio of activity limitations free life expectancy to life expectancy^1,5,19^.

In our analysis, we excluded outliers in *r* that fell outside of three standard deviations (SD) in the initial estimation, which might be due to a very low sample size within a particular sex-specific age group within a country. This was necessary for some observations of the 85+ population and the age group 80–84 (in total 11 observations out of 598). We identified a significant random variation in the *logit(r)* with likelihood ratio tests, which supported the applied random coefficient models. We run the following random coefficient model:

1 $${\mathtt{l}}{\mathtt{o}}{\mathtt{g}}{\mathtt{i}}{\mathtt{t}}({r}_{ic})={\beta }_{0}+{\beta }_{1}ag{e}_{ic}+{\beta }_{2}ag{e}_{ic}^{2}+{\beta }_{3}se{x}_{ic}+{\mu }_{0c}+{\mu }_{3c}se{x}_{ic}+{\varepsilon }_{ic}$$

considering a linear and quadratic effect of age on the *logit*(*r*); with *i* indicating the first level and *c* the country (second level). Further, following earlier research on European data^5,19^, our regressions did not consider changes of *r*_*ic*_ over time, however changes in life expectancy over time are accounted for by using life tables through 2050. Thus, the ratio of HLE to life expectancy was assumed to remain constant over time, which means that increases in life expectancy occur together with increases in HLE. In all countries investigated life expectancy is expected to increase. In a next step, we used the remaining life expectancies by age from the life tables provided by the United Nations^9^ to project HLE by sex, age, and country and prevalence of activity limitations subsequently. We estimated country, sex and age specific ${\bar{r}}_{ic}$ considering also the random variation by sex across countries. Next, we projected the share of older men and women with severe activity limitations from 2017 to 2047 for all 23 countries using sex specific population projections provided by the United Nations^9^.

Finally, we employed the constant characteristic age approach introduced by Sanderson and Scherbov^39^, which enabled comparisons on the very common age metric. Fixing 25% prevalence in severe activity limitations for each sex and country we estimated the age at which this level of prevalence was attained. These estimates were produced for 2017 and 2047.

## Data Availability

The World Health Survey (WHS) data that support the findings of this study are available from the Central Data Catalog of the World Health Organisation (http://apps.who.int/healthinfo/systems/surveydata/index.php/catalog/whs). UN World Population Prospects data can be obtained from the United Nations (https://population.un.org/wpp/Download/Standard/Population/).
